# Effects of plant nutrient acquisition strategies on biomass allocation patterns in wetlands along successional sequences in the semi-arid upper Yellow River basin

**DOI:** 10.3389/fpls.2024.1441567

**Published:** 2024-09-02

**Authors:** Xuan Wang, Le Wang, Weimin Li, Yifan Li, Yu An, Haitao Wu, Yue Guo

**Affiliations:** ^1^ State Key Laboratory of Black Soils Conservation and Utilization, Northeast Institute of Geography and Agroecology, Chinese Academy of Sciences, Changchun, China; ^2^ University of Chinese Academy of Sciences, Beijing, China; ^3^ Wetland Protection and Management Office of Jilin Province, Changchun, China

**Keywords:** biomass allocation, nutrient acquisition strategies, upper Yellow River basin, semi-arid wetlands, successional sequences

## Abstract

The ecological environment of wetlands in semi-arid regions has deteriorated, and vegetation succession has accelerated due to climate warming-induced aridification and human interference. The nutrient acquisition strategies and biomass allocation patterns reflect plant growth strategies in response to environmental changes. However, the impact of nutrient acquisition strategies on biomass allocation in successional vegetation remains unclear. We investigated 87 plant communities from 13 wetland sites in the semi-arid upper Yellow River basin. These communities were divided into three successional sequences: the herbaceous community (HC), the herbaceous–shrub mixed community (HSC), and the shrub community (SC). The nutrient composition of stems and leaves, as well as the biomass distribution above and belowground, were investigated. Results revealed that aboveground biomass increased with succession while belowground biomass decreased. Specifically, SC exhibited the highest stem biomass of 1,194.53 g m^−2^, while HC had the highest belowground biomass of 2,054.37 g m^−2^. Additionally, significant positive correlations were observed between leaf and stem biomasses in both HC and SC. The nitrogen (N) and phosphorus (P) contents within aboveground parts displayed an evident upward trend along the succession sequence. The highest N and P contents were found in SC, followed by HSC, and the lowest in HC. Stem N was negatively correlated with stem, leaf, and belowground biomass but positively correlated with root–shoot ratio. Leaf P displayed positive correlations with aboveground biomass while showing negative correlations with belowground biomass and root–shoot ratio. The ratios of C:N, C:P, and N:P in stem and leaf exhibited positive correlations with belowground biomass. The random forest model further demonstrated that stem N and leaf P exerted significant effects on aboveground biomass, while leaf P, stem N and P, and leaf C:P ratio had significant effects on belowground components. Additionally, the root–shoot ratio was significantly influenced by leaf P, leaf C:P ratio, and stem N, P, and C:P ratio. Therefore, the aboveground and belowground biomasses exhibited asynchronism across successional sequences, while plant nutrient acquisition strategies, involving nutrient levels and stoichiometric ratios, determined the biomass allocation pattern. This study offers valuable insights for assessing vegetation adaptability and formulating restoration plans in the semi-arid upper Yellow River basin.

## 1 Introduction

The succession of vegetation and the replacement of dominant plant species are common and crucial ecological processes that are manifest in the adaptation and development of plant communities under diverse environmental conditions (Graae et al., 2018; Zhang et al., 2020). The vegetation succession in wetlands characterized by dominant species turnover can be attributed to a combination of factors including soil hydrological and climate changes, human disturbance, and species invasion (Merino-Martín et al., 2012; Goodwillie et al., 2020; Zhang et al., 2022a; Ma et al., 2024). Climate change diminishes the adaptability of the original dominant species, thereby creating opportunities for the survival and reproduction of other species (Pätzig and Düker, 2021; Ren et al., 2021). Human disturbances such as urbanization, agricultural development, and pollution pose threats or alterations to wetland plant communities (Liang et al., 2021; Wang et al., 2022). Moreover, as time progresses and environmental conditions change, the succession processes within wetland ecosystems frequently entail the substitution of dominant species by new ones. The invasive species alter plant community composition by exerting competitive pressure on the original dominant species (Burge et al., 2017). For instance, shrub invasions in savanna wetlands nonlinearly modify both the structure and species composition of wetland vegetation (Barbosa Da Silva et al., 2016). The complex effects resulting from shrubs replacing herbs during succession have implications for vegetation’s adaptation and response to changing environments.

Along successional sequences, significant differences are observed in the growth environment, species composition, and ecological functions of plant communities (Geng et al., 2022), which are often closely associated with nutrient acquisition strategies (Barot et al., 2016; Zhang et al., 2017). Previous studies have shown that the nutrient distribution of different plant organs presents a consistent correlation, indicating a regularity in nutrient acquisition (Kerkhoff et al., 2006; Zhao et al., 2022). Wetland plants distribute resources to both aboveground and belowground parts, adapting to their habitats. This adaptation plays a pivotal role in regulating the survival and growth of plant communities (Fortunel et al., 2012). According to optimal partitioning theory, biomass distribution among different organs is adjusted by plants to adapt to resource availability and environmental pressures (Yin et al., 2019). Under this hypothesis, environmental changes and nutrient acquisition are driving forces behind biomass allocation between aboveground and belowground organs (Mccarthy and Enquist, 2007; Liu et al., 2021). Nutrient acquisition and biomass allocation, as key ecological processes influencing plant community structure and function during wetland vegetation succession, profoundly impact the stability and service function of wetland ecosystems (Kerkhoff et al., 2006; Hong et al., 2016). Therefore, it is crucial to fully consider the roles of nutrient acquisition and biomass allocation when studying mechanisms driving wetland vegetation succession (Melendez Gonzalez et al., 2019).

A well-developed plant root system can penetrate deeper into the soil to extract nutrients, which has a great impact on aboveground plant community growth and biomass allocation (Fan et al., 2017; Wu et al., 2022; Li et al., 2024). Plants obtain a more sufficient supply of nutrients, thereby promoting aboveground growth, including stem thickening and the exuberance of branches and leaves (Maciá-Vicente et al., 2022). In addition, a symbiotic relationship can be formed by some plants with specific microorganisms to obtain nutrients that are difficult to absorb directly. For example, most plants form a symbiotic relationship with arbuscular mycorrhizal fungi, which improves the utilization efficiency of nutrients (Huang et al., 2020). Higher nutrient utilization efficiency is typically observed in C_4_ photosynthetic plants compared to C_3_ plants, as limited nutrients are converted into the biomass required for growth through the employment of carbon content mechanisms and higher photosynthetic efficiency (Arce Cubas et al., 2023). Nutrient acquisition and biomass allocation in plants are optimized through strategies such as well-developed root systems, symbiotic relationships, and nutrient use efficiency, greatly influencing growth and environmental adaptation (Doležal et al., 2024).

However, ongoing climatic changes and unreasonable human activities have led to increasingly severe problems for the wetland ecosystem, including shrinking wetland areas, loss of biodiversity, and reduction in vegetation coverage (Liu et al., 2022; Fluet-Chouinard et al., 2023; Weiskopf et al., 2024). These changes not only lead dominant species to shift from herbaceous plants to shrubs but also profoundly impact the structure and functionality of wetland communities. During the process of vegetation succession, adapting to changes in nutrient acquisition strategies is essential to promoting the continuous development of the plant community. Different strategies for nutrient acquisition and utilization are adopted by different plant species, leading to diverse and dynamic patterns of biomass distribution (Abrahão et al., 2019; Wong et al., 2024). Particularly during vegetation degradation processes, biomass allocation patterns within alpine meadow plant communities of the Qinghai-Tibetan Plateau are gradually being altered by nutrient competition (Zhang et al., 2022b). However, research on how nutrient acquisition strategies of plant communities along the wetland succession gradient in the Yellow River upstream specifically affect biomass allocation remains limited, hindering a comprehensive understanding of the succession processes and functional characteristics of wetland ecosystems in semi-arid regions.

The upper Yellow River basin in Ningxia, located in northwest China’s semi-arid region, is crucial for its unique wetland ecosystems. Shaped by specific climatic conditions and abundant water resources, it plays a pivotal role in maintaining regional biodiversity and ecological balance (Wohlfart et al., 2016; Ji et al., 2023). In this study, we investigated and analyzed plant communities along the wetland succession sequence in the upper Yellow River basin, elucidating the relationship between nutrient acquisition and biomass allocation. We aim to explore the ecological mechanism by which nutrient acquisition influences biomass allocation under varying environmental conditions and analyze the key factors involved. Specifically, this research will provide a scientific basis for protecting and restoring wetland ecosystems in the upper Yellow River basin. Additionally, our findings offer valuable references and insights into understanding response mechanisms and adaptation strategies employed by semi-arid wetland ecosystems worldwide.

## 2 Materials and methods

### 2.1 Study area

**Figure 1:**
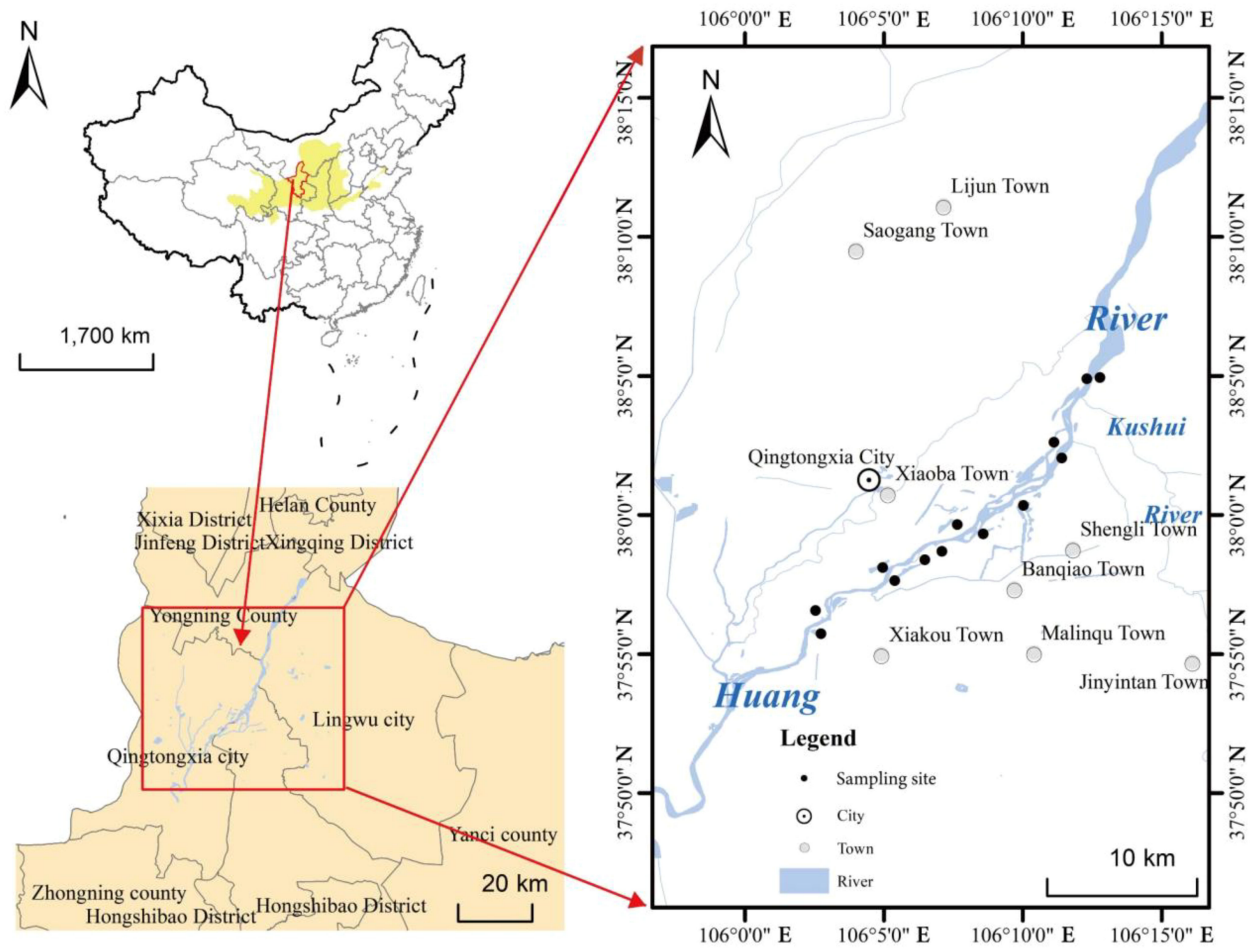
Location of study sites and selected sampling sites in the upper Yellow River basin.

This study was conducted in the upper Yellow River basin wetland located in Ningxia, China (**Figure 1**). The region exhibits a medium-temperate continental climate with an average annual precipitation of 289 mm, primarily concentrated in the summer. It is therefore significantly affected by seasonal precipitation and Yellow River flooding. The surface soil is mostly alkaline, containing gravel and sand (Liu et al., 2023). The average annual temperature ranges from 5.3°C to 9.9°C. This study area primarily encompasses both banks of the Yellow River (37°55′–38°05′N, 106°02′–106°12′E) and is characterized by its distinct natural vegetation community structure, shallow water table, and minimal geomorphological variation. Dominant herbaceous species within the study area include *Phragmites australis*, *Typha orientalis*, *Calamagrostis epigejos*, *Juncellus serotinus*, *Bolboschoenus planiculmis*, and *Polygonum hydropipe*. Additionally, notable shrub species consist of *Populus przewalskii*, *Populus alba*, *Salix integra*, and *Tamarix chinensis*. Climate change and human activities have disrupted the hydrological balance in this area, leading to accelerated succession of wetland herbaceous vegetation and subsequent invasion by shrubs.

### 2.2 Sample collection and analysis

#### 2.2.1 Field investigation and measurements

In this study, 13 representative wetlands located along the upper Yellow River banks were selected as sample sites in October 2023. Each sample site was divided into two or three sampling zones measuring 200 m long × 20 m wide. Within each sampling zone, three repeated quadrats measuring 1 m × 1 m were established. The entire sampling area consisted of a total of 29 plant community sampling zones, comprising a grand total of 87 quadrats, with each quadrat surrounded by a buffer zone ranging from 5 m to 15 m. Species within each quadrat were recorded to categorize them into three successional community types based on the distribution of herbaceous and shrubbery plants. They are herbaceous community (HC), herbaceous–shrub mixed community (HSC), and shrubbery community (SC).

The aboveground parts of the plants in each quadrat were harvested and further separated into stems and leaves. Correspondingly, roots were excavated from a soil layer that extended up to a depth of approximately 30 cm and subsequently washed with tap water for belowground biomass determination. The plant samples were dried at a constant temperature of 65°C until reaching a constant weight. The root–shoot ratio and biomass of aboveground, stem, leaf, and belowground were then quantified. The stem and leaf samples were pulverized for the analysis of organic carbon (C), nitrogen (N), and phosphorus (P). Organic carbon content was determined using an oxidative method involving a solution containing 0.4 mol L^−1^ of potassium dichromate (KCr_2_O_7_-H_2_SO_4_) followed by titration with 0.2 mol L^−1^ of FeSO_4_ (Niu et al., 2011). The N and P contents were measured using an automated chemical analyzer (Smartchem 300, Italy). Experimental measurements enabled the calculation of carbon-to-nitrogen (C:N), carbon-to-phosphorus (C:P), and nitrogen-to-phosphorus (N:P) ratios.

#### 2.2.2 Statistical analysis

The plant nutrient contents and ratios and biomass at different successional stages were subjected to statistical analysis using one-way analysis of variance (ANOVA) in SPSS. Prior to analysis, normality was tested using the Kolmogorov–Smirnov test, and homogeneity of variances was assessed using the Levene test. Differences among ANOVA variables were tested for significance using the least significant difference (LSD) test with a significance level of *p* < 0.05. The impact of plant nutrient acquisition on aboveground biomass, belowground biomass, and biomass partitioning was assessed by correlation analysis at *p* < 0.001, 0.01, and 0.05 levels, respectively. The random forest model can identify important variables for interpretation when there are strong interactions among plant nutrient variables at *p* < 0.01 and 0.05 levels. Data were analyzed and visualized using R 4.3.2 software (R Core Team, 2023).

## 3 Results

### 3.1 Biomass allocation pattern of wetland vegetation on succession sequences

**Figure 2:**
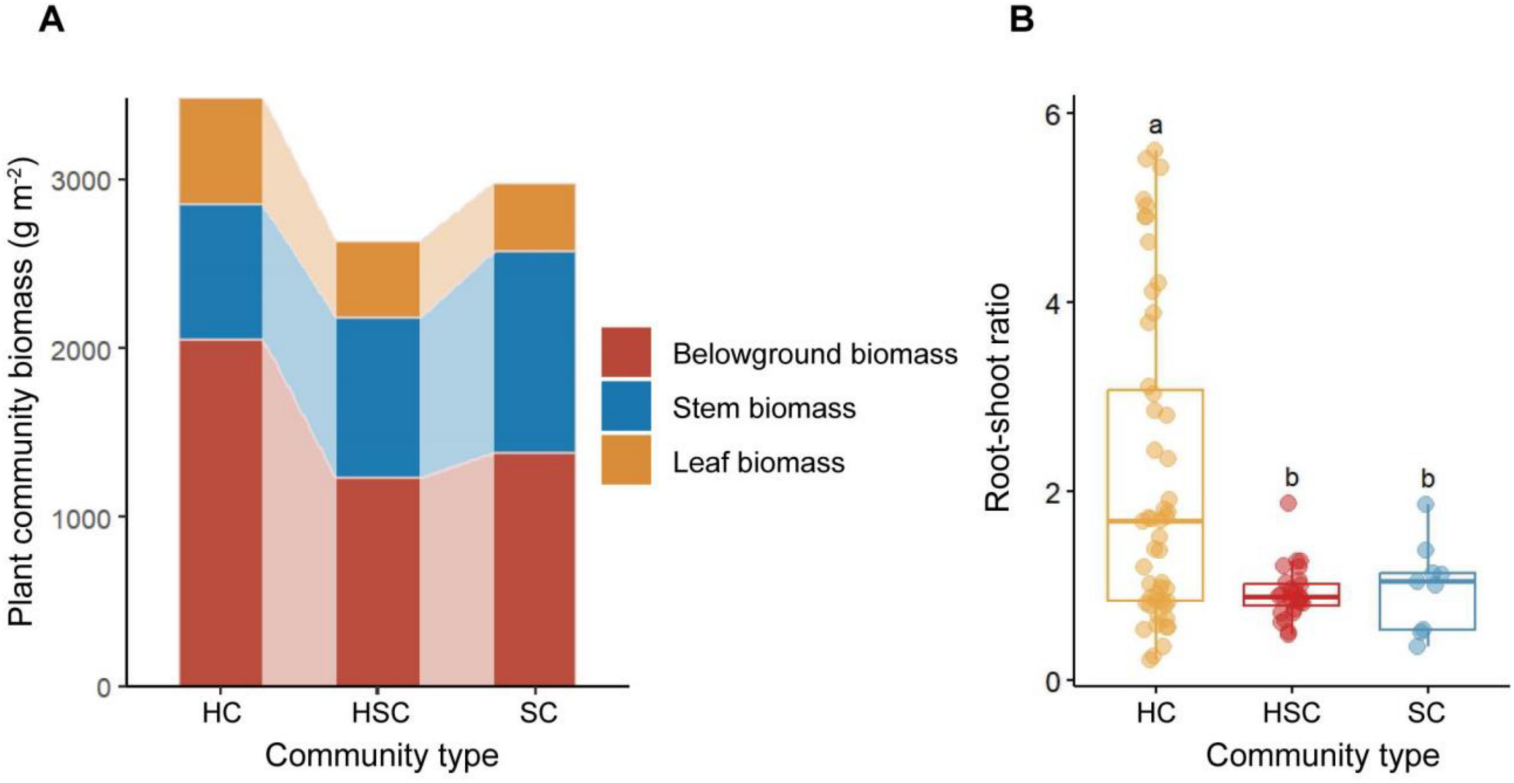
Patterns of belowground–stem–leaf biomass allocation **(A)** and root–shoot ratio **(B)** in HC, HSC, and SC. The significant difference in root–shoot ratio among plant communities was indicated by different letters (*p* < 0.05).

The biomass allocation patterns of HC, HSC, and SC along successional sequence in wetlands are shown in **Figures 2A, B**. HC exhibited a higher belowground biomass of 2,054.37 g m^−2^ (*p* < 0.05), a higher leaf biomass of 633.49 g m^−2^ (*p* > 0.05), and a lower stem biomass of 797.13 g m^−2^ than other plant communities (*p* > 0.05). The total biomass of HC was significantly greater than that of other plant communities (*p* < 0.05). The leaf biomass of HC, HSC, and SC exhibited a decrease along the successional sequence, while stem biomass increased. In terms of root–shoot ratio, HC displayed a significantly higher root–shoot ratio compared to HSC and SC (*p* < 0.05, **Figure 2B**). However, there was no significant difference in root–shoot ratios between HSC and SC (*p* > 0.05). Overall, the root–shoot ratio demonstrated a decreasing trend during the transition from HC to SC.

**Figure 3:**
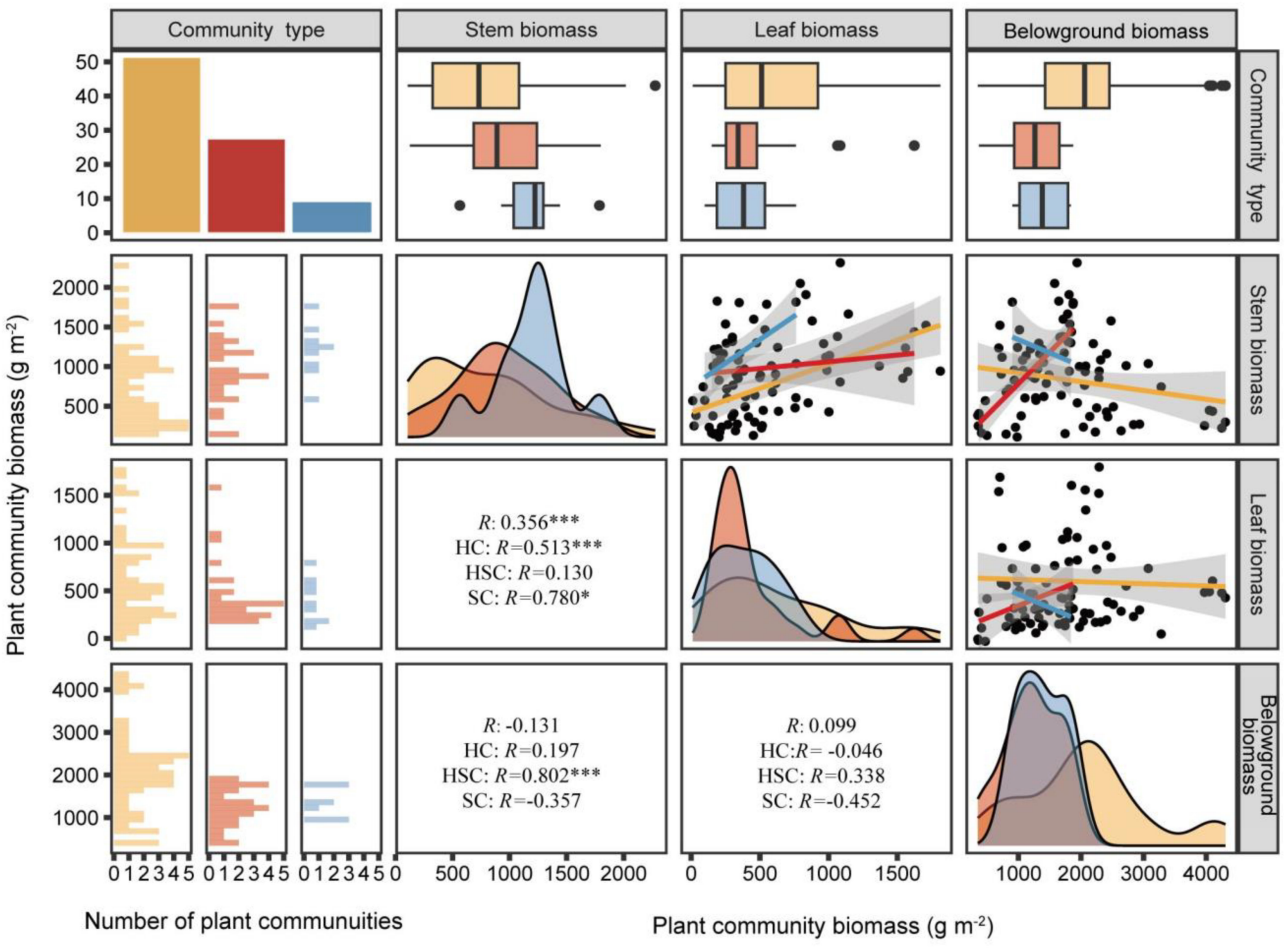
Relationships of belowground, stem, and leaf biomasses of HC, HSC, and SC. HC is represented by yellow, HSC by red, and SC by blue. *R* values represent Pearson’s correlations. ^*^0.05; ^***^0.001—levels of significance.

As shown in **Figure 3**, leaf biomass showed a significant positive correlation with stem biomass in HC (*p* < 0.001, *R* = 0.513) and SC (*p* < 0.05, *R* = 0.780), with the slope of the leaf–stem biomass relationship being steeper in SC. However, there was no significant relationship between stem and leaf biomass in HSC (*p* > 0.05). In HSC, stem biomass was positively correlated with belowground biomass (*p* < 0.001, *R* = 0.802). Conversely, the stem and leaf biomass of HC and SC did not show a significant relationship with their respective belowground biomass (*p* > 0.05).

### 3.2 Nutrient content of wetland vegetation on successional sequences

**Figure 4:**
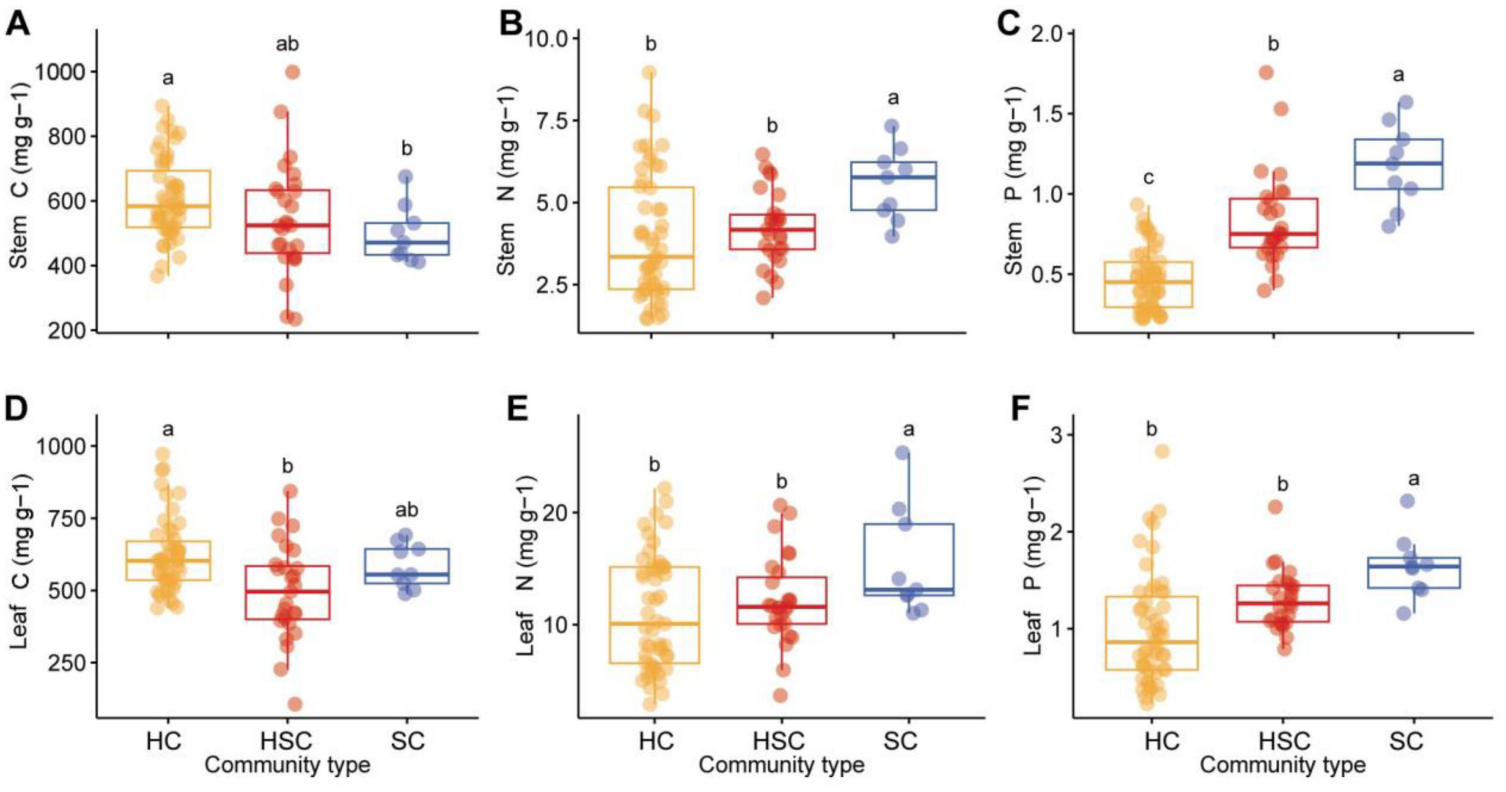
Comparison of C, N, and P contents of stem and leaf in HC, HSC, and SC. Significant differences in stem C **(A)**, stem N **(B)**, stem P **(C)**, leaf C **(D)**, leaf N **(E)**, and leaf P **(F)** among plant communities are indicated by different letters (*p* < 0.05).

The stem C content was significantly higher in HC compared to SC, while leaf C was also significantly higher in HC compared to HSC (*p* < 0.05 for all, **Figure 4**). However, there was no significant difference in stem C between HC and HSC and no significant difference in leaf C between HC and SC (*p* > 0.05 for all, **Figures 4A, D**). In **Figures 4B, C, E, F**, it is observed that nitrogen (N) and phosphorus (P) levels in stems and leaves of SC were significantly higher than those of HC and HSC (*p* < 0.05). Furthermore, N and P levels exhibited an increasing trend with successional sequence.

The stoichiometric ratios between stem and leaf C, N, and P of the wetland community along the successional sequence are summarized in **Table 1**. Leaf C:N, C:P, and N:P ratios, as well as stem C:P and N:P ratios, exhibited significantly higher values in HC than in HSC and SC (*p* < 0.05), indicating a decreasing trend of nutrient partitioning with succession. Specifically, HC had stem and leaf N:P ratios of 8.77 and 12.07, respectively, while for HSC and SC, these ratios were 5.49 and 9.62, and 4.83 and 9.37, respectively. The N:P ratios of all plant communities were observed to be less than 14, indicating a nitrogen-limited condition in this study area.

**Table 1:** Stem and leaf ecological stoichiometric characteristics and plant species in herbaceous, herbaceous shrub mixed, and shrub communities.

Community type	C:N		C:P		N:P		Plant species
	Stem	Leaf	Stem	Leaf	Stem	Leaf	
HC	193.09 ± 14.57 a	72.49 ± 6.21 a	1,524.05 ± 96.41 a	873.10 ± 95.40 a	8.77 ± 0.43 a	12.07 ± 0.47 a	*Phragmites australis*, *Typha orientalis*, *Calamagrostis epigejos*, *Juncellus serotinus*, *Bolboschoenus planiculmis*, and *Polygonum hydropiper*
HSC	137.24 ± 8.61 ab	45.24 ± 4.32 b	739.12 ± 67.88 b	397.96 ± 28.64 b	5.49 ± 0.39 b	9.62 ± 0.59 b	*Typha orientalis*, *Phragmites australis*, *Calamagrostis epigejos*, *Salix integra*, and *Tamarix chinensis*
SC	92.88 ± 10.07 b	40.72 ± 4.09 b	443.25 ± 47.97 b	368.37 ± 31.85 b	4.83 ± 0.30 b	9.37 ± 0.64 b	*Salix integra*, *Tamarix chinensis*, *Populus przewalskii*, and *Populus alba*

Significant differences in ecological stoichiometric characteristics (mean ± SE) among plant communities are indicated by different letters (*p* < 0.05).

### 3.3 Effect of nutrient content and stoichiometric ratios in stems and leaves on vegetation biomass allocation

**Figure 5:**
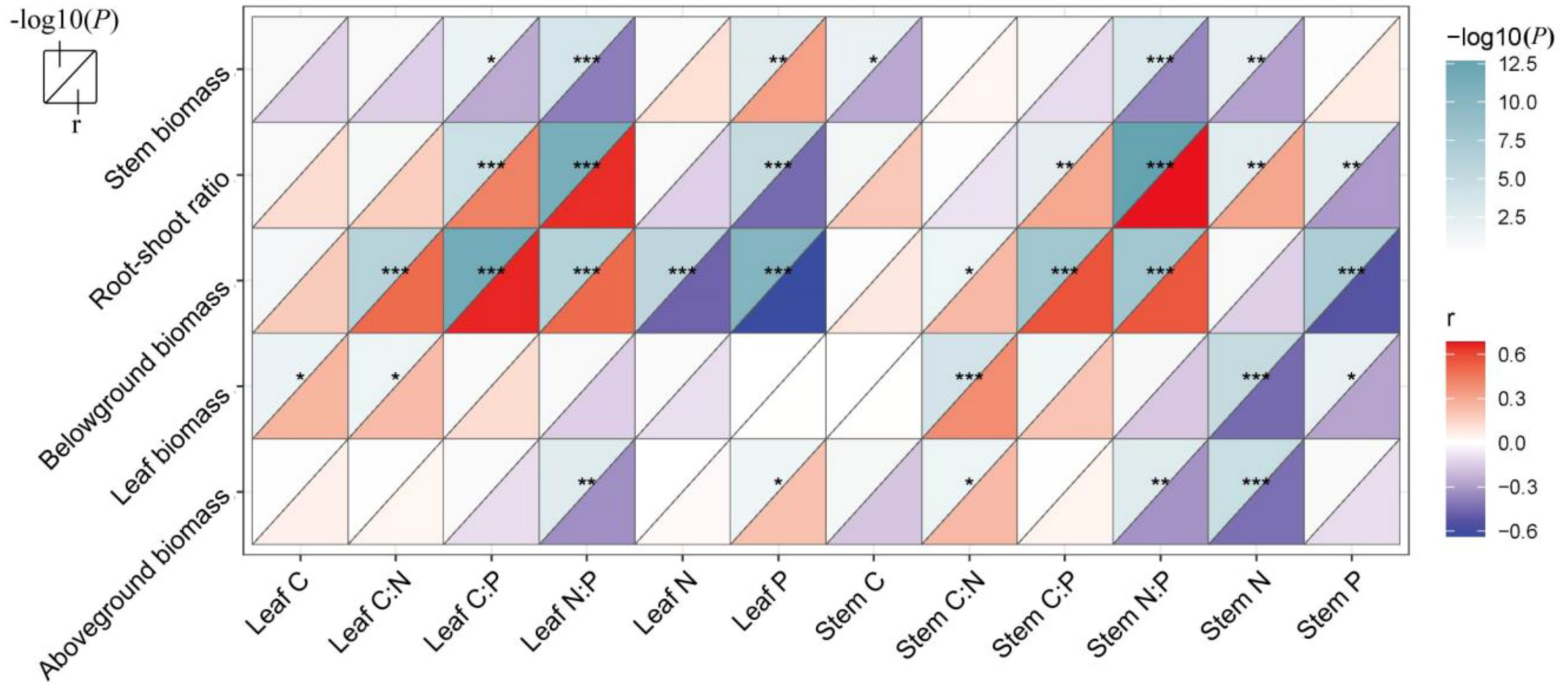
Correlations between nutrients in stem, leaves, and biomass. *p*-values (converted to −log10(*P*)) are shown in the figure to the upper triangle of each grid, and coefficients *r* are shown to the lower triangle of each grid. ^*^0.05; ^**^0.01; ^***^0.001—levels of significance.

**Figure 6:**
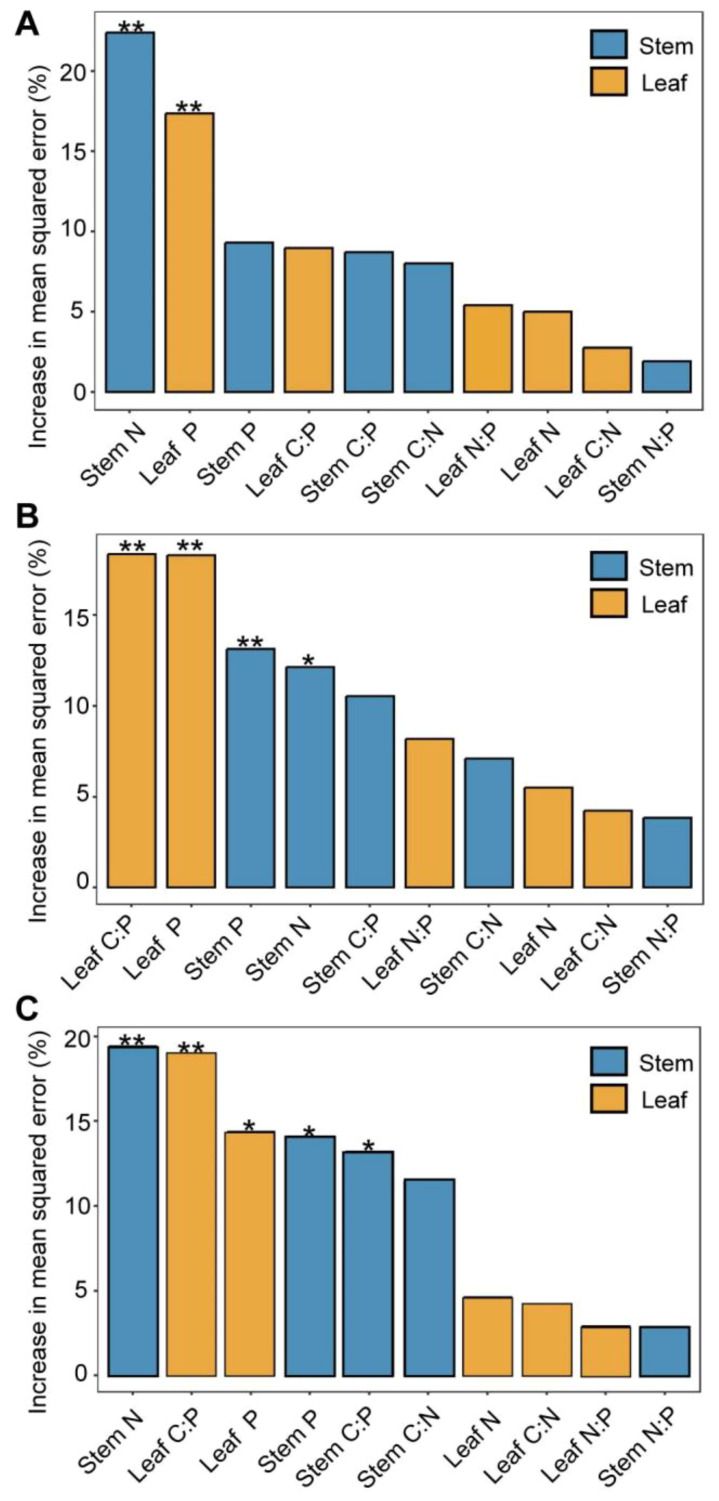
Effects of stem and leaf nutrients and their stoichiometric ratios on biomass. Importance of random forest mean predictive values for aboveground biomass **(A)**, belowground biomass **(B)**, and root–shoot ratio **(C)**. ^*^0.05; ^**^0.01—levels of significance.

There was a strong correlation between vegetation biomass allocation and the plant’s C, N, and P elements (**Figure 5**). Stem biomass showed a positive correlation with leaf P and a negative correlation with stem C, stem N, and N:P ratio and leaf C:P and N:P rations (*p* < 0.05). Leaf biomass exhibited a positive correlation with stem C:N ratio and leaf C and C:N ratio, while it was negatively correlated with stem N and P contents (*p* < 0.05). Additionally, the aboveground biomass showed a positive correlation with stem C:N and leaf P contents, while it exhibited a negative correlation with stem N, stem N:P, and leaf N:P ratios (*p* < 0.05). Belowground biomass, had positive correlations with both stem and leaf C:N:P ratios, but negative correlations with leaf N and P and stem P contents (*p* < 0.05). The root–shoot ratio demonstrated positive correlations with stem C:P ratio, stem N:P ratio, stem N, leaf C:P ratio, and leaf N:P ratio, while negatively correlating with stem P and leaf P (*p* < 0.01). Furthermore, results of random forest analysis showed that aboveground biomass was significantly influenced by stem N and leaf P (*p* < 0.01, **Figure 6A**). Belowground biomass was significantly influenced by leaf C:P ratio, leaf P, stem P, and stem N contents (*p* < 0.05, **Figure 6B**), with the plant community leaf nutrient factor accounting for a higher significance, and therefore belowground biomass was more influenced by community leaf nutrient content. Root–shoot ratio was significantly influenced by stem N, P, and C:P ratio and leaf P and C:P ratio (*p* < 0.05 for all, **Figure 6C**).

## 4 Discussion

### 4.1 Changes in vegetation biomass on successional sequences

At the early stage of the successional sequence, a greater proportion of biomass in HC is allocated to structures utilized for resource acquisition, such as plant roots. Conversely, as the successional sequence progresses, herbaceous plants are replaced by shrubs, and more biomass is allocated to structural organs (Stevens et al., 2017; Zhang et al., 2023a). Shrub species tend to invest more resources into stem growth compared to herbaceous plants, facilitating taller plant size and structure (Urbina et al., 2020). Simultaneously, the increase in stem biomass results in additional resources being allocated to leaf development by plants. This enhances photosynthesis and improves nutrient uptake from an ecological perspective (Sun et al., 2019). Consequently, aboveground biomass exhibits an upward trend along the successional sequence. Furthermore, this study reveals a negative correlation between aboveground biomass in HSC and belowground biomass. The mixed community comprising both herbs and shrubs represents a stage characterized by shrub invasions where root systems may not yet be fully established (Zhang et al., 2019; Yu et al., 2022). Belowground biomass also increases, but usually does not match the growth of the aboveground portion, as the root system is gradually expanded and deepened by shrubs (Shen et al., 2022; Zhang et al., 2022b). This study found that the relationship of synchronous growth between aboveground biomass and belowground biomass was disrupted, which we speculate is influenced by the encroachment of shrubs. It is evident that shrubs exhibit higher aboveground biomass compared to herbaceous plants, while the increase in belowground biomass is not synchronous. This indicates that while aboveground biomass increases, belowground biomass may not increase simultaneously and could even decrease, and thus the root–shoot ratio decreases.

The regulation of root–shoot ratios reflects the ability of plants to adapt to environmental changes, enabling them to better cope with variations in water and nutrient availability across different habitats (Mašková et al., 2022). This study finds that the biomass allocation pattern of vegetation is influenced by shrub invasions, as evidenced by the disruption of the synergistic balance between stem and leaf growth in HSC (D’Odorico et al., 2012). Additionally, we found significant positive correlations between leaf and stem biomasses in HC and SC, suggesting a synergistic growth pattern. However, this synergy was disrupted by shrub invasions in HSC. This result is probably attributed to differentiated resource acquisition strategies, ecological niche differentiation, root structure variations, and growth habits (Zuppinger-Dingley et al., 2014; Stefanowicz et al., 2016). This study revealed that HC exhibited higher root–shoot ratios, suggesting a more developed belowground system compared to their aboveground counterparts. Herbaceous plants tend to produce thinner roots while maintaining a certain level of belowground biomass, thereby optimizing resource acquisition from the soil through an increased root–shoot ratio and enhancing their environmental adaptability (Roumet et al., 2008; Rojas-Botero et al., 2023). Moreover, it was observed that the vegetation biomass of herbs was transferred and distributed more through carbon sinks than that of shrubs (Guo et al., 2022).

### 4.2 Variations of aboveground nutrient content and stoichiometric ratios on successional sequences

Vegetation at different successional stages exhibits distinct nutrient acquisition strategies, with early succession characterized by high nutrient requirements and metabolic growth rates, while that with late succession shows more vigorous growth (Rondina et al., 2019). The nutrient content of stems and leaves varies across vegetation types and successional stages, generally increasing gradually as succession progresses. In this study, it is noted that N and P contents in SC are the highest, followed by HSC and HC. On the one hand, many shrubs that invade grasslands, such as *Tamarix chinensis*, can engage in symbiotic nitrogen fixation, thereby increasing the nitrogen content at the community level (Sitters et al., 2013; Araya et al., 2022). On the other hand, shrubs have deeper root systems than herbs, allowing them to obtain phosphorus from soils that herbs cannot (Jackson et al., 1996; Zhou et al., 2018b). In the early stages of succession, herbaceous plants occupy the dominant position within the community, which may simultaneously exhibit a high nutrient content in their leaves but a relatively low nutrient content in their stems. As succession progresses, shrubs become dominant, and there is a gradual increase in the nutrient content of both stems and leaves (Smith and Read, 2008). The N and P contents in stems and leaves increased with succession sequence in this study, reflecting the adjustment of nutrient distribution in plants to adapt to growth and ecological requirements at different succession stages.

The stoichiometric composition of plant tissues can be altered by community composition and nutrient interactions, which occur during succession and shrub expansion (Sistla and Schimel, 2012; Zhou et al., 2018a). It was reported that the C:N:P stoichiometric ratios in plant tissues differ among life types, such as grasses, forbs, or shrubs (Peng et al., 2011; Di Palo and Fornara, 2017). Significant decreases in community C:N, C:P, and N:P ratios were observed along the successional sequence of herb, herb–shrub, and shrub in this study. This change is likely indicative of a shift in the roles and functions of different types of plants in the ecosystem. For example, in the initial stages, more carbon and nitrogen may be accumulated in soil by herbaceous plants for their growth and survival, whereas in subsequent stages, the expansion of shrubs may lead to a redistribution of carbon and nitrogen in soil, resulting in a decrease in the C:N, C:P, and N:P ratios of plant tissues.

### 4.3 Influence of vegetation nutrient acquisition strategies on biomass allocation

During the process of growth, plant communities balance resource allocation and spatial competition to develop habitat-adapted biomass allocation patterns (Yin et al., 2019; Ma and Wang, 2021). A higher nitrogen or phosphorus content implies that plants acquire nitrogen and phosphorus nutrients more efficiently from the soil. We assume that this particular plant community employs superior nutrient acquisition strategies compared to other plant communities, enabling them to fully absorb and utilize nitrogen and phosphorus, thereby increasing their growth rate and biomass accumulation capacity. Generally, an increase in nutrient levels usually has a positive effect on plant growth; however, beyond a certain threshold level, this effect may become negative (Güsewell, 2005; Zhang et al., 2021). It is contrary to our expectations that we observed a negative correlation between nitrogen contents in stems and leaves and community biomass. This finding suggests that there is not a simple facilitative relationship between plant community biomass and vegetation nutrient level (Ding et al., 2023). Moreover, both stem and leaf P exerted notable effects on the root–shoot ratio, displaying a prominent negative correlation in this study. This suggests that plants regulate their nutrient utilization strategies to maintain ecosystem stability. As nitrogen and phosphorus contents increase in stems and leaves, there is a preference for utilizing these nutrients for aboveground growth and maintenance at the expense of investment in the root system (Oldroyd and Leyser, 2020). Consequently, N and P contents in stems and leaves may rise while belowground biomass decreases (Gruber et al., 2013).

The community N:P ratio can serve as an indicator of nitrogen and phosphorus limitation. In this study, the N:P ratio of plant communities exhibited a decreasing trend along the successional sequence, consistently remaining below 14. This finding is consistent with previous research showing that nitrogen serves as the primary limiting factor for vegetation growth (Koerselman and Meuleman, 1996). However, excessive nitrogen levels can result in imbalanced growth and have a detrimental impact on overall plant development (Zhang et al., 2023b). To ensure the healthy ecological development of wetlands, we emphasize reducing external nutrient inputs from agricultural runoff or wastewater discharge. In this study, we observed a positive correlation between leaf C:P at the community level and belowground biomass. Additionally, N and P in stem and leaf showed significant negative correlations with belowground biomass, indicating that nutrient content and ratios in plant leaves directly influence belowground biomass (Ma et al., 2020; Zadworny et al., 2021). In this study, there was an observed decrease in community leaf C:P ratios along successional sequences. The decrease in the leaf C:P ratio implies an increase in carbon demands and a reduction in the photosynthetic efficiency of plants (Hu et al., 2021; Xiao et al., 2024). Consequently, as photosynthetic efficiency decreases, it is expected that plants will produce less organic carbon, leading to a decrease in belowground biomass. Additionally, due to heightened carbon demand in leaves, there may be a reduced allocation of organic carbon to the community root system, with more resources directed toward fulfilling leaf growth requirements. This adjustment in resource allocation strategy is anticipated to result in diminished belowground biomass and lower root–shoot ratio.

## 5 Conclusion

This study investigates the vegetation’s nutrient acquisition strategy and its impact on biomass allocation in a successional sequence of semi-arid wetlands in the upper Yellow River basin. Differential responses are observed between aboveground and belowground biomasses along a successional sequence regarding their nutrient acquisition strategies. Notably, the aboveground parts show continuous change, yet the root systems do not synchronize therewith. Specifically, there is a phenomenon of delayed belowground biomass production during shrub invasions. Furthermore, as the community N:P ratio decreases, nitrogen limitation becomes influential and affects aboveground growth; however, leaf nutrients promote root development resulting in a decreasing trend of root-shoot ratio followed by a slow rebound along with shrub invasions. The significance of vegetation nutrient acquisition and utilization is underscored for distinguishing among herbs, herb–shrub mixes, and shrubs concerning both nutrient partitioning and biomass allocation, particularly evident due to wetland ecosystems’ vulnerability. Understanding how nutrient variation in wetland plants affects biomass allocation patterns during succession improves predictions of wetland ecological dynamics in semi-arid regions. This knowledge informs effective conservation and management strategies in response to changing environments, emphasizing crucial vegetation nutrient acquisition and utilization strategies to enhance ecological resilience and mitigate environmental impacts.

## Data Availability

The original contributions presented in the study are included in the article/supplementary material. Further inquiries can be directed to the corresponding author.
